# Evaluation of Sleep Quality in Asthmatic Children with the Paediatric Sleep Questionnaire (PSQ)

**DOI:** 10.3390/children11060728

**Published:** 2024-06-14

**Authors:** Mehmet Yaşar Özkars, Seda Çevik, Serap Ata, Alican Sarısaltık, Uğur Altaş

**Affiliations:** 1 Department of Pediatric Allergy and Immunology, Ümraniye Training and Research Hospital, University of Health Sciences, 34764 Ümraniye, Türkiye; seda.gul@saglik.gov.tr (S.Ç.); ugur.altas@saglik.gov.tr (U.A.); 2 Department of Pediatrics, Ümraniye Training and Research Hospital, University of Health Sciences, 34764 Ümraniye, Türkiye; serap.ata@saglik.gov.tr; 3 Çayırova District Health Directorate, 41420 Çayırova, Türkiye; alican.sarisaltik@marmara.edu.tr

**Keywords:** sleep-disordered breathing disorders, sleep quality, children, asthma, Paediatric Sleep Questionnaire

## Abstract

Considering the high prevalence of sleep-related breathing disorders (SRBD) in asthmatic patients, we aimed to compare asthmatic children and healthy children in terms of SRBD according to Paediatric Sleep Questionnaire (PSQ) scores. A questionnaire covering sociodemographic characteristics of the patients and the PSQ, which evaluates sleep quality and consists of 22 questions, was administered. During the data collection process, 180 patients in the patient group and 170 patients in the control group were included. The patient group showed statistically significantly higher total scores and subscale scores for snoring, sleepiness, and inattention compared to the control group. Statistically significant correlations were found between the sleepiness subscale and body mass index z score in a negative direction and between age at presentation and duration of asthma in a positive direction. Our findings endorse employing the PSQ as a screening instrument in the outpatient environment to ensure timely referral of asthma patients to a sleep specialist for SRBD evaluation. Considering the widespread occurrence of snoring and asthma, this tool could aid in identifying patients with an elevated risk of SRBD and expedite the scheduling of nocturnal polysomnography for these children.

## 1. Introduction

Obstructive sleep apnoea (OSA) and its spectrum of symptoms are part of sleep-related breathing disorders (SRBD) that impact various facets of a child’s life and development negatively [1]. Obstructive sleep apnoea is the most common type of sleep-disordered breathing, affecting approximately 1% of children, and may lead to problems including excessive daytime sleepiness, behavioural problems, learning difficulties, growth retardation, heart failure, or failure to thrive [2,3,4]. While adenotonsillar hypertrophy and allergic rhinitis are the primary causes of SRBD in children, neuromuscular tone pathology is also implicated in its aetiology [5,6]. The known risk factors for OSAS include tonsillar and adenoid hypertrophy, obesity, craniofacial anomalies, neuromuscular disorders, and African-American ancestry [7]. Childhood obstructive OSA is often linked to symptoms such as snoring, episodes of apnoea, shortness of breath, and recurrent infections of the upper airway [8].

Sleep laboratory based polysomnography (PSG) remains the benchmark for diagnosing and assessing OSA in children [1]. However, PSG is an expensive technique and may not be easily available in many cases [9]. In addition to PSG, drug-induced sleep endoscopy (DISE) is a diagnostic procedure used to evaluate the upper airway of patients with OSA under conditions that closely simulate natural sleep. This technique helps identify the specific sites and patterns of airway obstruction, thereby guiding appropriate treatment strategies [10]. Conversely, sleep surveys serve as practical and cost-effective screening instruments for both primary care paediatricians and counsellors addressing children’s sleep issues. The Paediatric Sleep Questionnaire (PSQ), crafted and authenticated by Chervin et al. [11], is extensively employed for SRDB screening in both research and clinical environments [12,13]. It was adapted to the Turkish population by Yüksel et al. [14].

The prevalence of SRBD is higher in asthmatic patients compared with non-asthmatic children [15,16]. In a forward-looking investigation, children with asthma who experience SRBD were found to be 3.62 times more prone to severe asthma during a twelve-month monitoring period [17]. Furthermore, children with inadequately managed asthma face an increased likelihood of severe (OSA). Managing OSA through adenotonsillectomy could enhance asthma management by decreasing asthma related flare-ups and the usage frequency of rescue inhalers [18]. Consequently, it is crucial to screen children with uncontrolled asthma for SRBD, enabling prompt referral for formal OSA evaluation.

Considering the higher prevalence of SRBD in asthmatic patients, we aimed to evaluate the SRBD in asthmatic children and healthy children according to PSQ scores. The evaluation of the correlations between BMI, asthma duration, age, and PSQ total and sub-scores were our secondary objectives, since these factors may affect sleep in asthmatic patients.

## 2. Materials and Methods

### 2.1. Study Type and Population

In this case-control study, paediatric patients aged 2–18 years with a diagnosis of asthma who presented to our Paediatric Allergy and Immunology Clinic were included as the patient group. A control group was formed from the children aged 2–18 years who came to the same hospital’s paediatric health and diseases outpatient clinic for vaccination services, well-child check-ups, and routine examinations. Children without sleep disorders such as asthma or OSA or other chronic diseases were included in the control group. Both the control and patient groups excluded children under the age of two, those diagnosed with sleep disorders, and those with psychiatric diseases or medication use that could affect sleep. The minimum sample size was calculated as 185 patients by accepting the frequency of sleep disturbance in children with asthma as 14.5%, with margin of error as 5% and confidence level as 95% [19]. It was planned to include at least 185 children in the healthy control group. During the data collection process, 180 patients in the patient group and 170 patients in the control group were included.

### 2.2. Evaluation

Sociodemographic characteristics of the patients, such as age at diagnosis and gender, and responses to the Paediatric Sleep Questionnaire (for ages 2–18 years), which evaluates sleep quality and consists of 22 questions, were collected via questionnaire. Anthropometric measurements (height, weight, and BMI z scores), family history for asthma, asthma duration, presence of allergic rhinitis, and presence of gastroesophageal reflux were also asked. Asthma control was evaluated according to the Global Initiative for Asthma (GINA) criteria and asthma patients were divided into three groups: well controlled, partly controlled, and uncontrolled [20]. All these factors are considered as confounding factors for sleep quality; for this reason, we asked about these factors and evaluated the relationship with PSQ scores.

The Paediatric Sleep Questionnaire (PSQ) comprises 22 questions categorised into three symptom clusters: snoring, excessive daytime sleepiness, and hyperactive or inattentive behaviour. A cumulative score exceeding 0.33 (indicating positive responses to 33% of the questions) suggests SRDB, with an 81% sensitivity and 87% specificity in the overall paediatric cohort [11]. When contrasted with alternative sleep surveys validated for paediatric use, the PSQ demonstrates superior validity in OSA detection [12,13].

### 2.3. Statistical Analysis

Statistical analysis was performed using IBM SPSS Statistics for Windows software (Version 22.0; Armonk, NY, USA, IBM Corp.) Kolmogorov–Smirnov and Shapiro–Wilk tests were used to examine the conformity of continuous data to normal distribution. Median and interquartile range (IQR) values were used for continuous variables. The Mann–Whitney U test was used to analyse the relationships between individual characteristics and PSQ total and subscale scores. The total PSQ scores of the participants were categorised by accepting 0.33 as the cut-off value. Chi-square and Fisher’s exact test were used to analyse categorical variables. The correlations between individual characteristics and PSQ total and subscale scores were evaluated with Spearman’s rank correlation coefficient. In the analyses, *p* < 0.05 was accepted as the statistical significance level.

## 3. Results

In our research, the median age at initial presentation was 6.92 years among patients and 8.29 years among controls (*p* = 0.123). The percentage of males was 59.4% in the patient group (*n* = 107) and 49.4% in the control group (*n* = 84) (*p* = 0.060). There were no significant distinctions between the groups regarding age, gender, height, weight, and BMI z scores (*p* = 0.123, *p* = 0.060, *p* = 0.531, *p* = 0.177, and *p* = 0.308, respectively). The median duration of asthma in the patient group was 12 months and the disease was well controlled in more than half of the group (*n* = 102, 56.7%). Thirty-four patients had a family history of asthma (18.9%). Approximately three-quarters of the patients had allergic rhinitis as a comorbidity (*n* = 138, 76.7%) (Table 1).

The patient group showed statistically significantly higher total scores and subscale scores for snoring, sleepiness, and inattention compared to the control group (*p* < 0.001 for all) (Table 2). Similarly, the proportion of patients with a PSQ total score of 0.33 and above was 1.2% (*n* = 2) in the control group and 47.8% (*n* = 86) in the patient group, and the difference between the groups was statistically significant (*p* < 0.001) (Table 2).

The correlations between individual and medical characteristics and the total score and subscale scores of the PSQ in the patient group are presented in Table 3. Statistically significant low positive correlations were found between the total score of the PSQ and age at presentation and duration of asthma (rho = 0.203 and rho = 0.177, respectively). Additionally, statistically significant but low correlations were observed between the sleepiness subscale and BMI z score in a negative direction and between age at presentation and duration of asthma in a positive direction (rho = −0.194, rho = 0.300 and rho = 0.175, respectively).

In the patient group, the proportion of men with a PSQ total score ≥0.33 was statistically considerably higher than that of women (55.1% and 37.0%, respectively, *p* = 0.017). In studying the connection between comorbidity and sleep quality, a patient with reflux was evaluated in the same category as those with allergic rhinitis for statistical evaluation. Although the frequency of patients with asthma and allergic rhinitis or reflux comorbidities who had a PSQ total score ≥0.33 was higher than those without comorbidities, the difference among the two groups was not significant (50.4% and 39.0%, respectively, *p* = 0.202). No significant correlation was found between asthma severity and the presence of asthma in the family and PSQ scores (Table 4).

## 4. Discussion

The incidence of SRBD, encompassing a broad spectrum of symptoms spanning from snoring to OSA, has been documented as low as 2% for OSA and as elevated as 27.6% for snoring [6,21,22,23]. SRBD holds clinical and epidemiological significance due to its impact not only on physical growth but also on emotional development, quality of life, and neurocognitive functions in children [24,25,26,27,28]. Recent research indicates that children diagnosed with asthma are more likely to persist with severe OSA necessitating continuous positive airway pressure (CPAP) therapy compared to their counterparts without asthma [29]. In our study, it was found that the PSQ scores of our case group with asthma were significantly higher than those of the healthy control group. In our study, the rate of patients with a PSQ total score of 0.33 and above was 1.2% in the control group and 47.8% in the patient group. 

While recent reviews have outlined the effect of asthma on sleep [16,30], it is important to highlight some key aspects here. The prevalence of uncontrolled or severe asthma among paediatric asthma patients ranges from 5% to 10% [31,32,33]. In our study, uncontrolled asthma was observed in 2.2% of the patients. No significant difference was found between the PSQ scores of uncontrolled patients and well-controlled patients. This may be due to the small number of patients with uncontrolled asthma. Children experiencing severe and inadequately managed asthma encounter sleep disturbances [34,35], such as frequent awakenings and reduced sleep efficiency caused by REM-related respiratory issues [36,37]. Frequent asthma symptoms may result in daytime sleepiness and fatigue, impacting daytime performance and cognitive functions [38,39].

Obesity is a risk factor for both asthma and OSA [40,41,42]. The presence of obesity may have an effect on PSQ scores. BMI scores were also identified in our study. The significant negative correlation between sleepiness and BMI z score in our study may be attributed to the effect of other confounding factors. There are various hypotheses about the relationship between OSA and severe asthma. Nocturnal asthma could result in sleep disturbances, potentially amplifying upper airway collapse (a contributing factor for OSA) [37,43]. However, the possible correlation between asthma and sleep-disordered breathing still needs to be investigated. For instance, a previous investigation did not find a correlation between OSA severity and asthma in children [44]. 

Moreover, a significant portion of individuals with asthma and OSA suffer from both non-allergic and allergic rhinitis. Rhinitis may induce alterations in nasal airway resistance (and consequently airflow rate), causing an elevation in upper respiratory negative pressure, thereby intensifying upper airway collapse and triggering symptoms of SRBD [45,46]. Allergic rhinitis was found in 76.7% of our case group. Rhinitis can induce alterations in nasal air passage resistance (and consequently airflow speed), resulting in an elevation of negative pressure in the upper airway, amplifying upper airway collapse and leading to symptoms of SRBD [45,46]. In addition to this, some studies in the literature have reported that sleep disruption in asthmatic patients is attributed to the aggravation of asthma by GER events [47,48]. Although the frequency of patients with asthma with allergic rhinitis or reflux comorbidity who had a PSQ total score ≥0.33 was higher in patients with asthma than in patients without comorbidity, the difference between the two groups was not found to be significant. Further studies are needed to better investigate the impact of comorbidities on sleep in asthmatic patients.

### Limitations and Strengths

The number of patients with poorly controlled asthma was small, potentially limiting the power of the comparisons made. Future studies should aim to recruit a larger sample of poorly controlled asthma patients to validate these findings more robustly. Gastroesophageal reflux disease (GERD) can significantly influence the severity of sleep disturbances. Thus, another limitation of our study was the lack of consideration of the presence of GERD in participants. Another limitation of the study was the lack of data on medication compliance or pulmonary function. The presence of OSA, one of the most common disorders negatively affecting sleep quality in children, was not assessed in our study. Ideally, given that polysomnography is the gold standard for the diagnosis of obstructive sleep apnoea, polysomnographic data should be used to evaluate sleep disorders. The lack of these data is another limitation of this study. The study is conducted in a specific geographic and clinical setting, which may limit the generalizability of the findings to other populations. However, we consider the Ümraniye district to be a moderately developed sample that can represent the socio-demographic and economic characteristics of Türkiye’s population [49].

Despite its limitations, our study makes a significant contribution to the literature through its strengths. We have a comprehensive evaluation of sleep quality in asthmatic children using the PSQ, alongside a control group of healthy children. By analysing the correlations between BMI, asthma duration, age, and PSQ scores, we provide insights into factors affecting sleep in asthmatic patients. Our study highlights the need for routine sleep screening and focused interventions, thus significantly contributing to the field of paediatric sleep medicine and asthma management.

## 5. Conclusions

In our study, the patient group showed statistically significantly higher total scores and subscale scores for snoring, sleepiness, and inattention compared to the control group. Higher PSQ scores were seen in older children and children with longer duration of asthma.

Our findings endorse employing the PSQ as a screening instrument in the outpatient environment to ensure timely referral of asthma patients to a sleep specialist for SRBD evaluation. Considering the widespread occurrence of snoring and asthma, this tool could aid in identifying patients with an elevated risk of SRBD and expedite the scheduling of nocturnal polysomnography for these children. This implies that employing the PSQ during the evaluation of asthmatic children with uncontrolled symptoms, irrespective of their weight status, will assist clinicians in deciding whether additional assessment for sleep apnoea or adjusting asthma treatment is necessary.

## Figures and Tables

**Table 1:** Individual and medical characteristics of the participants.

	Participant Groups		*p*-Value
	Control Group (*n* = 170)	Asthma Patients (*n* = 180)	
Male gender	84 (49.4)	107 (59.4)	0.060 *
Age at presentation (years)	8.29 (5.67–11.67)	6.92 (5.50–9.96)	0.123 **
Height Z score	0.31 (−0.41–1.04)	0.25 (−0.69–1.13)	0.531 **
Weight Z score	0.49 (−0.31–1.21)	0.38 (−0.54–1.09)	0.177 **
BMI Z score	0.43 (−0.43–1.34)	0.33 (−0.98–1.15)	0.308 **
Comorbidity			
None	-	41 (22.8)	-
Allergic rhinitis	-	138 (76.7)	
Reflux	-	1 (0.6)	
Allergic rhinitis + reflux	-	0 (0.0)	
Duration of asthma (months)	-	12.0 (3.0–36.0)	-
Asthma control			
Well controlled	-	102 (56.7)	-
Partly controlled	-	74 (41.1)	
Uncontrolled	-	4 (2.2)	
Asthma in the family	-	34 (18.9)	-

BMI: Body mass index, *n* (%) and median (interquartile range) values are shown. * Chi-square test; ** Mann–Whitney U test.

**Table 2:** Comparison of the Paediatric Sleep Questionnaire scores of the participant groups.

	Participant Groups		*p*-Value
	Control Group, Median (IQR)	Asthma Patients, Median (IQR)	
PSQ total score	0.00 (0.00–0.45)	0.32 (0.16–0.45)	<0.001 *
Snoring	0.00 (0.00–0.00)	0.25 (0.00–0.25)	<0.001 *
Sleepiness	0.00 (0.00–0.00)	0.25 (0.00–0.50)	<0.001 *
Inattention	0.00 (0.00–0.00)	0.33 (0.00–0.66)	<0.001 *
Total score ≥ 0.33; *n* (%)	2 (1.2)	86 (47.8)	<0.001 **

IQR: Interquartile range; PSQ: Paediatric Sleep Questionnaire; * Mann–Whitney U test; ** Chi-square test.

**Table 3:** Correlations between anthropometric measurements, age, asthma duration, and PSQ scores in the patient group (*n* = 180).

	Total Score	Snoring	Sleepiness	Inattention
Height Z score	−0.103	0.019	−0.146	0.029
Weight Z score	−0.068	0.018	−0.133	−0.100
BMI Z score	−0.135	−0.043	−0.194 *	−0.099
Age at presentation (years)	0.203 *	−0.087	0.300 **	0.083
Duration of asthma (months)	0.177 *	0.056	0.175 *	0.135

Spearman’s rho values are shown. PSQ: Paediatric Sleep Questionnaire. * *p* < 0.05; ** *p* < 0.01.

**Table 4:** Relationships between individual and medical characteristics and PSQ scores in the patient group (*n* = 180).

		PSQ Total Score		*p*-Value
		<0.33; *n* (%)	≥0.33; *n* (%)	
Gender	Female	46 (63.0)	27 (37.0)	0.017 *
	Male	48 (44.9)	59 (55.1)	
Comorbidity	None	25 (61.0)	16 (39.0)	0.202 *
	Allergic rhinitis/reflux	69 (49.6)	70 (50.4)	
Asthma control	Well controlled	53 (52.0)	49 (48.0)	0.999 **
	Partly controlled	39 (52.7)	35 (47.3)	
	Uncontrolled	2 (50.0)	2 (50.0)	
Asthma in the family	No	78 (54.3)	68 (46.6)	0.503 *
	Present	16 (47.1)	18 (52.9)	

PSQ: Paediatric Sleep Questionnaire; * Chi-square test; ** Fisher–Freeman–Halton Exact test.

## Data Availability

The original contributions presented in the study are included in the article, further inquiries can be directed to the corresponding author.
